# Ovarian Yolk Sac Tumor in a 10‐Year‐Old Girl Following Metachronous Bilateral Mature Ovarian Teratomas

**DOI:** 10.1155/crog/9281498

**Published:** 2026-10-13

**Authors:** Shiho Yoshida, Sakika Shimizu, Keisuke Kato, Geoffrey J. Lane, Go Miyano, Toshihiro Yanai

**Affiliations:** ^1^ Department of Pediatric Surgery Ibaraki Children′s Hospital Mito Japan; ^2^ Pediatric General and Urogenital Surgery Juntendo University School of Medicine Bunkyo Tokyo Japan juntendo.ac.jp; ^3^ Department of Pediatric Hematology and Oncology Ibaraki Children′s Hospital Mito Ibaraki Japan

**Keywords:** mature teratoma, ovarian germ cell tumor, ovarian torsion, yolk sac tumor

## Abstract

**Background:**

Ovarian germ cell tumors are the most common ovarian malignancies in children. Because these patients have a notable risk of developing metachronous bilateral tumors or recurrences, maximizing the preservation of ovarian tissue during initial surgery is critical to protecting future fertility and endocrine function.

**Objective:**

A previously healthy 8‐year‐old girl underwent emergency laparotomy for ovarian torsion, resulting the right oophorectomy, with histopathology confirming a mature teratoma. Sixteen months later, a contralateral ovarian mass was detected and treated with ovarian‐sparing excision, also diagnosed as a mature teratoma. At age 10 (10 months after the second excision), she was found to have an 8 cm pelvic tumor and elevated *α*‐fetoprotein. Magnetic resonance imaging (MRI) showed complex solid and cystic components suggestive of a malignant germ cell tumor, with no metastases on computed tomography/MRI. The mass was removed intact at exploratory laparotomy, yielding a diagnosis of Fédération Internationale de Gynécologie et d′Obstétrique (FIGO) Stage 1A ovarian yolk sac tumor. She was discharged after completing combination chemotherapy. Currently, she is 16 years old and being managed with hormone replacement therapy.

**Conclusions:**

The presented case highlights a disadvantage of overzealous oophorectomy. Detorsion of the seemingly necrotic ovary followed by second‐look surgery could have preserved some ovarian function.

## 1. Introduction

Ovarian tumors in children aged up to 15 years old occur at a rate of 2.6 per 100,000 girls annually, making them relatively rare [1]. However, from a pediatric oncology perspective, germ cell tumors are the most common type of pediatric surgical malignancy in all children, with ovarian primaries accounting for 102/161 cases (63%) in 2017 in Japan [2]. Ovarian tumors in girls are benign mature teratomas in 57%–77%, immature teratomas in 11%, and yolk sac tumors in 3% of cases, and 8.1% of girls with ovarian tumors developed metachronous diseases or tumor recurrences [2, 3]. Although approximately 70% of metachronous tumors are also benign mature teratomas, recurrences frequently occur in immature teratomas arising from remnant ovarian tissue, representing around 5% of ovarian tumors after ovarian‐sparing tumor excision [3].

Torsion of the ovary commonly complicates an ovarian tumor by the ovary twisting on its supporting ligaments, compromising vascular perfusion. Timely detorsion could save an ovary, but oophorectomy may be inevitable if necrosis of an ovary is too advanced, and the ovary is considered nonviable. Otherwise, treatment of an ovarian tumor typically involves ovarian‐sparing surgery as a single procedure. However, if there is metachronous onset of another tumor in the contralateral ovary after oophorectomy performed for any reason, or malignant transformation of remnant ovarian tissue, preserving ovarian function becomes challenging. This is particularly relevant in young girls because any deterioration in ovarian function will negatively impact growth, physical development, and fertility. As such, preservation of ovarian function is a crucial consideration when treating ovarian tumors.

Here, a case of ovarian yolk sac tumor that arose in remnant ovarian tissue after ovarian‐sparing surgery for a mature teratoma of the left ovary that developed after a mature teratoma of the right ovary caused torsion and necrosis that required oophorectomy.

## 2. Case Presentation

A previously healthy 8‐year‐old Japanese girl presented to our emergency department with 2‐day history of worsening, persistent abdominal pain. Abdominal ultrasonography (US) revealed a 7 × 8 cm pelvic cystic mass with calcification. Subsequent computed tomography (CT) suggested right ovarian torsion with severe organ enlargement (Figures 1a,b). At emergency laparotomy, a 540‐degree torsion of an ischemic right ovary was identified (Figure 1c). Despite attempted ovarian‐sparing surgery, normal ovarian tissue could not be distinguished from the tumor, and a right oophorectomy was performed. The contralateral left ovary appeared normal upon inspection and palpation. The patient had an uneventful postoperative recovery and was discharged on Day 4. Histopathological examination confirmed a benign mature teratoma (Figure 1d), and routine follow‐up with US was scheduled.

**Figure 1:**
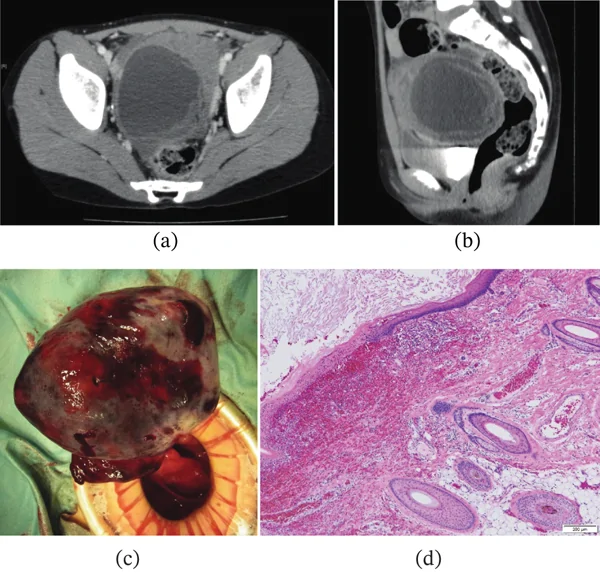
Diagnostic imaging and operative findings at the first operation (oophorectomy of the right ovary). (a,b) Abdominal contrast‐enhanced computerized tomography scan. (a) Coronal section and (b) sagittal section. A cystic mass (70 × 80 mm) occupying the pelvis is present. There is no enhancement of the tumor wall, and ovarian torsion was suspected. (c) Intraoperative findings. The right ovary was torted 540 degrees with a bluish‐black appearance. Even after detorsion, there was no improvement in color. The ovary was considered to be necrotic and right oophorectomy was performed. (d) Typical histology (hematoxylin and eosin staining; 10x magnification). No immature tissue was present. Mature teratoma was diagnosed.

Sixteen months after oophorectomy, a 1.6 × 1.4 cm mass was identified in the remaining left ovary on routine follow‐up US. Contrast‐enhanced CT and magnetic resonance imaging (MRI) suggested the mass was either a mature teratoma or dermoid cyst (Figure 2a,b). Given the previous right oophorectomy, a left ovarian‐sparing excision was promptly performed to maximize tissue preservation. Histopathological evaluation confirmed a second benign mature teratoma (Figures 2c,d). The postoperative course was unremarkable, and regular surveillance was resumed using the same follow‐up protocol.

**Figure 2:**
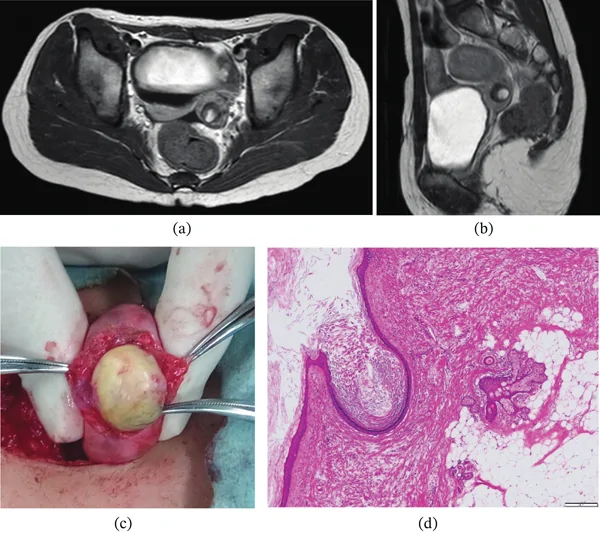
Diagnostic imaging and operative findings at the second operation (ovarian‐sparing surgery, left ovary). (a,b) Abdominal contrast‐enhanced MRI T1‐weighted image. (a) Transverse section and (b) sagittal section. A 1.6 × 1.4 cm tumor was observed in the left ovary. (c) Intraoperative findings. A well‐demarcated tumor was confirmed in the left ovary and was excised sparing normal ovarian tissue. (d) Histology (hematoxylin and eosin staining; 10x magnification). The resected tumor measured 17 × 15 mm and was examined in four tissue blocks. The tumor consisted of a simple cyst containing mature tissues, including epidermis, keratin debris, hair, adipose tissue, sebaceous glands, and glandular epithelium. No immature or malignant components were identified, and a mature teratoma was diagnosed.

Ten months after the ovarian‐sparing surgery (26 months after oophorectomy), routine follow‐up US detected a pelvic tumor, and laboratory testing revealed a markedly elevated alpha‐fetoprotein (AFP) level of 52,600 ng/mL (normal < 40 ng/mL). The patient was immediately admitted. On admission, she was asymptomatic, well‐developed, and in no acute distress. On physical examination, a smooth, poorly mobile mass was palpated occupying the entire lower abdomen with no palpable lymphadenopathy. Other tumor markers were within normal limits, including *β*‐human chorionic gonadotropin (< 0.1 ng/ml), lactate dehydrogenase (337 U/L), and CA125 (14.1 U/ml). Other blood tests (complete blood count, biochemistry, and coagulation panels) were unremarkable. Abdominal US identified a 10 × 6 cm solid mass compressing the bladder, with scattered small cysts and internal hypervascularity on Doppler (Figures 3a,b). Contrast‐enhanced MRI demonstrated a well‐defined, 7.6 × 8.4 × 8.7 cm pelvic mass with mixed cystic‐solid components, irregular enhancement, and restricted diffusion, without peritoneal thickening or lymphadenopathy (Figures 3c,d). Given the high suspicion of a malignant germ cell tumor, a left oophorectomy was performed via a Pfannenstiel incision. Intraoperatively, the left ovary was fist‐sized with an intact capsule and no evidence of torsion, ascites, or peritoneal dissemination (Figure 3e). Macroscopic examination of the resected specimen revealed a well‐encapsulated, purely solid tumor measuring 11 × 8 × 6 cm with no cystic components. Peritoneal lavage cytology was negative for malignancy. Histopathological evaluation demonstrated an ovarian yolk sac tumor, characterized by a prominent reticular/microcystic pattern and the presence of Schiller–Duval bodies (Figure 3e–g). These highly characteristic histomorphological findings supported the diagnosis, allowing for the prompt initiation of definitive treatment.

**Figure 3:**
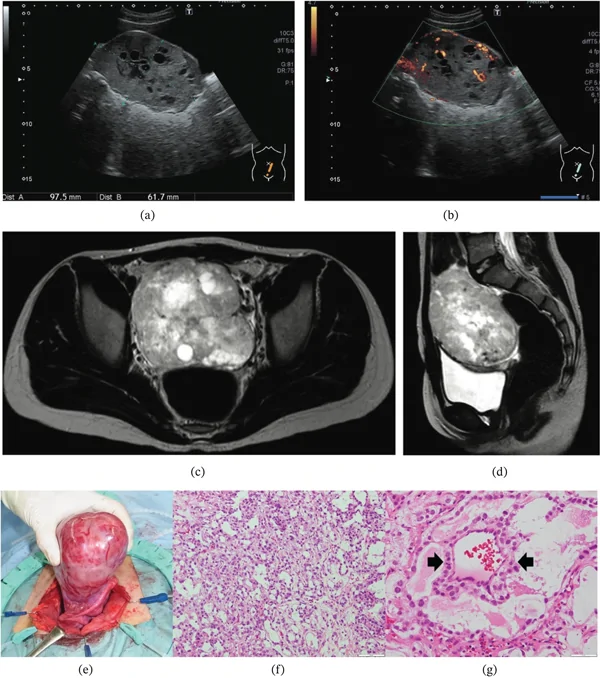
Diagnostic imaging and operative findings at the third operation. (a) Ultrasound imaging findings and (b) ultrasound color Doppler image. A 10 × 6 cm solid mass compressing the bladder was detected in the pelvis. (b) There is increased blood flow within the mass. (c,d) Contrast‐enhanced MRI T2‐weighted image. (c) Transverse section. (d) Sagittal section. Small cysts are seen scattered throughout the mass. A smooth and well‐defined mass (7.6 × 8.4 × 8.7 cm) was observed in the pelvis. The mass contained both cystic and solid components, showing irregular enhancement effects and diffusion impairment. (e) Intraoperative findings. A fist‐sized left ovarian tumor is visualized. The tumor capsule was completely intact, with no evidence of rupture, macroscopic intraperitoneal dissemination, or invasion into adjacent pelvic organs. (f,g) Histology. (Hematoxylin and eosin staining; (f) 20x magnification, (g) 40x magnification), (f) Reticular pattern. Tumor cells form a lace‐like network of spaces (microcysts) lined by flattened cells. (g) A Schiller–Duval body (black arrows), a characteristic glomerulus‐like structure consisting of a central blood vessel surrounded by tumor cells.

Based on the Fédération Internationale de Gynécologie et d’Obstétrique (FIGO) staging system, the patient was diagnosed with a Stage 1A ovarian yolk sac tumor. Postoperatively, she completed four cycles of BEP (bleomycin, etoposide, and cisplatin) chemotherapy. Because of the bilateral oophorectomies, hormone replacement therapy was initiated sequentially, starting with estradiol monotherapy and later transitioning to cyclic Kaufman therapy. Six years after her last surgery, the patient remained well with appropriate growth and no clinical or radiographic evidence of tumor recurrence or metastasis.

## 3. Discussion and Conclusions

The incidence of metachronous contralateral tumors after ovarian tumor surgery in girls is reported to be 3.2%, occurring on average 16.5 months (range 12–32 months) after the initial surgery [3]. A systematic review of females aged < 21 years old with mature ovarian teratomas reported an overall prevalence of metachronous contralateral mature teratoma of 2.1%, rising to 7% among studies conducting regular US follow‐ups for 3–5 years [4]. In the presented case, a metachronous contralateral ovarian tumor was detected 16 months after initial oophorectomy during routine follow‐up with US, emphasizing the importance of regular imaging surveillance. The recurrence rate for ovarian mature cystic teratoma after surgical removal is reported to be 3%–4%, and patients with recurrence were significantly younger than patients without recurrence [5]. Recognized risk factors for recurrence were being under 26 years of age, having one or no pregnancies, and being nulliparous [5]. Another study on ovarian tumors in girls under 16 years old reported a recurrence rate of 4.8%, with a median period of 12.5 months (range 6–15.5 months) after the initial surgery [3]. Data about recurrence has practical application for counseling about prognosis and providing background information to ensure thorough understanding of the pathology present. Ultimately, mature teratomas can also undergo malignant transformation. Although 0.8%–3.8% of mature ovarian teratomas transform into squamous cell carcinoma [6], pediatric populations are uniquely prone to primitive germ cell malignancies; for instance, 5.4% of resected sacrococcygeal teratomas develop into metachronous yolk sac tumors [7], and a sequential or concurrent development of yolk sac tumors alongside mature teratomas has been well‐documented in intracranial or periovarian tissues [8–10].

In the contemporary histopathological evaluation of such germ cell malignancies, immunohistochemical (IHC) analysis provides important diagnostic support. Yolk sac tumors typically express AFP, SALL4, and glypican‐3, whereas markers such as OCT3/4 and CD30 can aid in the differential diagnosis of other germ cell tumors. In our patient′s case, an IHC panel was not performed because the hematoxylin and eosin morphology showed highly characteristic features of a yolk sac tumor, including a prominent reticular pattern and Schiller–Duval bodies, in the setting of a markedly elevated serum AFP level. Nevertheless, the absence of IHC confirmation is a limitation of this report, as immunophenotypic evaluation would have provided additional diagnostic support and helped exclude other germ cell tumor components.

To understand the pathogenesis of this malignancy, the embryological origin of these tumors must be considered. Ovarian germ cell tumors arise from primordial germ cells (PGCs) that fail to undergo proper epigenetic reprogramming and differentiation during their migration along the embryonic hindgut to the developing gonads [11]. When these displaced or arrested PGCs retain their latent pluripotency, they can give rise to benign teratomas containing tissues from all three germ layers. However, under specific microenvironmental or genetic triggers, these improperly reprogrammed cells can undergo malignant somatic transformation or clonal selection, redifferentiating into primitive extraembryonic structures such as a yolk sac tumor [11].

In the presented case, three potential pathogenetic mechanisms may be considered for the rapid development of the yolk sac tumor 10 months after the left ovarian‐sparing surgery. First, the malignancy may represent malignant transformation of residual benign teratoma tissue following the previous surgery. Second, a previously undetected microscopic focus of yolk sac tumor may have coexisted within the secondary mature teratoma but been missed on initial histopathological sampling. Given that yolk sac tumors grow aggressively, even a minute occult component could potentially have developed into a large clinical mass postoperatively. Third, the tumor could have arisen de novo in the residual ovarian tissue, independently of the previous teratoma. Although the patient′s clinical history of sequential, bilateral teratomas raises the possibility of an association between these tumors, the available findings cannot distinguish among these three potential mechanisms.

Given the potential risk for metachronous contralateral tumors and the importance of preserving future fertility, ovarian‐sparing surgery should be considered a first‐line approach in pediatric patients when oncologically and technically appropriate. However, radical oophorectomy is not uncommon in emergency settings based on macroscopic appearance alone. A retrospective study by Spinelli et al. reported that oophorectomy was performed in up to 50.5% of cases of ovarian torsion in patients under 19 years old [12]. However, a study showed that many ovaries removed due to suspected necrosis from ovarian torsion contained viable tissue [13]. Additionally, there was no correlation between the macroscopic appearance of a torted ovary and future follicular development [14].

Therefore, when ischemia is suspected based on the purplish/bluish‐black appearance of a torted ovary, a conservative, multistage surgical approach is strongly indicated. Emergency intervention should be limited to detorsion, with or without cystectomy or oopexy [15]. Definitive tumor separation and tissue viability confirmation should then be deferred to a planned second‐look surgery, typically scheduled 1 week to 1 month later once the acute inflammatory phase has resolved [16]. This staged management safely maximizes ovarian tissue preservation, provides an optimal window to counsel families, and minimizes the risk of intraoperative tumor capsule spillage.

In conclusion, the authors strongly recommend avoiding radical oophorectomy based solely on macroscopic or ischemic appearance. A planned, multistage surgical approach should be actively utilized to preserve future fertility in young patients. Furthermore, because metachronous or recurrent disease may be difficult to predict at the time of initial surgery, even if the initial histopathology was benign, continued postoperative surveillance may facilitate early detection of secondary disease. Postoperative surveillance, including regular US scanning with consideration of periodic serum AFP monitoring, may be warranted. However, the optimal surveillance interval and duration remain to be established.

**Nomenclature**
*β*‐HCG: *β*‐human chorionic gonadotropin
AFP: alpha‐fetoprotein
CT: computerized tomography
FIGO: Fédération Internationale de Gynécologie et d′Obstétrique
IHC: immunohistochemical
LDH: lactate dehydrogenase
MRI: magnetic resonance imaging
PGCs: primordial germ cells.
US: ultrasonography

## Author Contributions

S.Y.: writing—original draft, methodology, investigation, conceptualization. S.S.: resources, methodology, investigation. K.K.: methodology, investigation. G‐J.L.: writing—review and editing. G.M.: validation, supervision. T.Y.: conceptualization, validation, supervision.

## Funding

No funding was received for this manuscript.

## Ethics Statement

The authors have nothing to report.

## Consent

Written informed consent was obtained from the patient for publication of this case report and accompanying images.

## Conflicts of Interest

The authors declare no conflicts of interest.

## Data Availability

The data that support the findings of this study are available on request from the corresponding author. The data are not publicly available due to privacy or ethical restrictions.
